# Antimicrobial resistance in commensal *Escherichia coli* isolated from animals at slaughter

**DOI:** 10.3389/fmicb.2013.00221

**Published:** 2013-08-05

**Authors:** Dariusz Wasyl, Andrzej Hoszowski, Magdalena Zając, Krzysztof Szulowski

**Affiliations:** National Reference Laboratory for Antimicrobial Resistance, Department of Microbiology, National Veterinary Research InstitutePuławy, Poland

**Keywords:** *Escherichia coli*, commensal, indicator bacteria, antimicrobial resistance, resistance trends, resistance phenotypes

## Abstract

Monitoring of antimicrobial resistance in commensal *Escherichia coli* (*N* = 3430) isolated from slaughtered broilers, laying hens, turkeys, swine, and cattle in Poland has been run between 2009 and 2012. Based on minimal inhibitory concentration (MIC) microbiological resistance to each of 14 tested antimicrobials was found reaching the highest values for tetracycline (43.3%), ampicillin (42.3%), and ciprofloxacin (39.0%) whereas the lowest for colistin (0.9%), cephalosporins (3.6 ÷ 3.8%), and florfenicol (3.8%). The highest prevalence of resistance was noted in broiler and turkey isolates, whereas it was rare in cattle. That finding along with resistance patterns specific to isolation source might reflect antimicrobial consumption, usage preferences or management practices in specific animals. Regression analysis has identified changes in prevalence of microbiological resistance and shifts of MIC values. Critically important fluoroquinolone resistance was worrisome in poultry isolates, but did not change over the study period. The difference (4.7%) between resistance to ciprofloxacin and nalidixic acid indicated the scale of plasmid-mediated quinolone resistance. Cephalosporin resistance were found in less than 3.8% of the isolates but an increasing trends were observed in poultry and MIC shift in the ones from cattle. Gentamycin resistance was also increasing in *E. coli* of turkey and cattle origin although prevalence of streptomycin resistance in laying hens decreased considerably. Simultaneously, decreasing MIC for phenicols observed in cattle and layers isolates as well as tetracycline values in *E. coli* from laying hens prove that antimicrobial resistance is multivariable phenomenon not only directly related to antimicrobial usage. Further studies should elucidate the scope of commensal *E. coli* as reservoirs of resistance genes, their spread and possible threats for human and animal health.

## Introduction

Bacterial resistance to antimicrobials has always been intriguing academic communities. Although high-tech methods are being available still there are gaps in understanding how bacteria can cope with chemicals, how they develop resistance strategies and transfer them between bacterial cells, species, or hosts. Some of the crucial restrictions are complexity of microbiota present in various ecosystems (Allen et al., 2013; Perchec-Merien and Lewis, 2013) and studies focused on selected issues at a given point of time (Dahmen et al., 2012; Wasyl et al., 2012). Human mobility and easy transfer of goods in globalized world further tangles up epidemiology of resistance (SVARM, 2012).

Resistant bacteria can compromise public and animal health and lead to severe economic losses in animal production (Morfin-Otero et al., 2012; Tadesse et al., 2012). Antimicrobial resistance affecting humans might develop either in public health area or food production chain where it can be transmitted from resistant bacteria to humans via several pathways, including direct contact with animals or indirect transfer *via* foods (EFSA, 2009). Monitoring of resistance in bacterial pathogens such as *Salmonella* aims at the timely counter-actions to combat life or welfare threatening infections (Morfin-Otero et al., 2012). Eradication, hygiene, and public awareness have diminished the major epidemiological threats. Subsequently, the number of pathogens available for antimicrobial resistance testing becomes insufficient. Commensal bacteria such as *E*. *coli*, give an alternative (Tadesse et al., 2012; Allen et al., 2013). Although there are some pathogenic phenotypes (Pitout, 2012), most of randomly selected *E. coli* from human or animal gut flora might be considered as non-pathogenic indicators for antimicrobial resistance (Kaesbohrer et al., 2012; Tadesse et al., 2012). The advantages of resistance monitoring in commensals result from their high prevalence, simple and efficient isolation procedures (Allen et al., 2013) and, compared to pathogenic bacteria, limited possibility for clonal spread.

Methods used for resistance detection should fit into the purpose of the testing. Novel molecular techniques offering optimal sensitivity for detection and characterization of resistance mechanisms, might be cost and time-consuming for routine application in clinical settings (Van Der Bij et al., 2012). Finding of resistance determinant in a bacterium might not correspond to its phenotypic resistance and *vice versa* (Ozaki et al., 2011; Wasyl et al., 2012). Therefore, classical microbiological methods are preferred for monitoring purposes (De Jong et al., 2012; EFSA, 2012b; EFSA and ECDC, 2012; Tadesse et al., 2012). No matter what method is used, resistance data need to be carefully interpreted. Clinical breakpoints are useful for assessment of probability of therapeutic success in clinical situation (Morfin-Otero et al., 2012; Van Der Bij et al., 2012). Epidemiological approach is appropriate for monitoring of pathogens and commensal bacteria, since it early detects the changes in resistance (EFSA, 2008). Evaluation of resistance trends in bacteria might be improved with quantitative data analyses (EFSA, 2012a; Van Der Bij et al., 2012).

Increase in antimicrobial resistance have led to enhanced world-wide studies on clinical aspects of resistance and its monitoring in both pathogens and commensal bacteria being potential reservoir of transmissible resistance determinants and indicator of antimicrobial use (EFSA, 2008, 2012b; EFSA and ECDC, 2012; Tadesse et al., 2012; Van Der Bij et al., 2012; Allen et al., 2013; Schroeter et al., 2013). Country-wide antimicrobial resistance monitoring in commensal *Escherichia coli* has been implemented in Poland since 2009 (Wasyl et al., 2010, 2012). Herewith, the four-year results are shown with special attention to the temporal trends analysis (EFSA, 2012a).

## Materials and methods

Antimicrobial resistance monitoring of indicator *E. coli* has been implemented in broilers, laying hens, turkey, cattle, and swine slaughtered in Poland.

### Sampling

On January the numbers of animals slaughtered over the previous year in each abattoir in the country were reported by veterinary service. Slaughterhouses contributing to the substantial national annual production were designated for sampling in the upcoming year. Two hundred samples per studied animal population were assigned for sampling evenly distributed over the upcoming 12 months. Characteristics of national production of slaughter animals, numbers of slaughterhouses and sampling plan criteria were shown in Table 1. Each sample, consisting of three rectal or cloacal swabs, was taken by veterinary officers from three consecutive, random animals from slaughter line immediately after slaughter. Transport medium cotton swabs were submitted to the laboratory via courier service and proceeded within a week after collection. A minimum set of information on the date, location and sample source was collected and submitted electronically to the central database designed for managing of the project.

**Table 1:** **Descriptive characteristics of studied populations relevant for sampling planning, number of samples collected and *E. coli* isolates used in the study**.

**Characteristics**	**Target animal**	**Year**				
		**2008**	**2009**	**2010**	**2011**	**2012**
Number of animals slaughtered in Poland	Broilers	554846197	578970522	633640413	666313527	723764340
	Layers	29550379	26353151	33890429	37311263	39056653
	Turkeys	27244356	22926577	25339326	25287042	27780566
	Swine	19525920	17420288	19488804	20038278	20094157
	Cattle	1601569	1492360	1580467	1523384	1578773
Number of slaughterhouses in Poland	Broilers	178	159	160	156	155
	Layers	51	44	43	40	37
	Turkeys	37	34	29	26	24
	Swine	912	706	694	682	662
	Cattle	440	355	349	351	340
Slaughter capacity threshold^*^	Broilers	3650000	2200000	2100000	2 200 000	
	Layers	70000	100000	50000	30 000	
	Turkeys	700000	250000	100000	90 000	
	Swine	19300	35000	39000	45 000	
	Cattle	3600	4750	4800	4 950	
Number of abattoirs designated for sampling and their contribution to annual slaughter capacity (%)^**^	Broilers	47 (80%)	61(85%)	66 (90%)	63 (90%)	
	Layers	7 (50%)	20 (95%)	20 (99%)	27 (99%)	
	Turkeys	15 (80%)	21 (95%)	23 (99%)	22 (99%)	
	Swine	200 (80%)	109 (70%)	94 (70%)	81 (70%)	
	Cattle	91 (80%)	79 (80%)	78 (80%)	72 (80%)	
Number of samples collected and proceeded	Broilers		180	186	192	195
	Layers		162	194	181	179
	Turkeys		180	188	194	189
	Swine		181	176	189	194
	Cattle		174	179	176	191
Number of tested *E.coli* isolates	Broilers		171	170	170	171
	Layers		157	169	155	157
	Turkeys		173	170	170	180
	Swine		178	170	172	190
	Cattle		173	171	173	190

* *Minimal number of animals slaughtered at abattoir to consider designation of slaughterhouse for sampling*.

** *Estimated contribution (%) of designated abattoirs to annual national production of slaughtered animals*.

### Laboratory testing

Swabs were streaked directly on MacConkey agar. Colonies showing typical *E. coli* morphology were confirmed biochemically and a single isolate representing each sample was selected for antimicrobial resistance testing with microbroth dilution method (Sensititre, TREK D. S.). Minimal Inhibitory Concentration (MIC) of 14 antimicrobials (Table 2) representing beta-lactams and cephalosporins, quinolones and fluoroquinolones, phenicols, aminoglycosides, folate-path inhibitors, tetracyclines and polymyxins were interpreted according to epidemiological criteria (EUCAST, www.eucast.org). Non-wild type (NWT) MICs above the cut-off values were classified as microbiological resistance and the isolate was supposed to carry relevant resistance determinant. Only phenotypic resistance was considered and resistance patterns were used to give possible insight into co-resistance and cross-resistance. Multi-drug resistance (MDR) was defined with the profile comprising at least one agent in three or more antimicrobial classes, extensive drug resistance (XDR)—from at least seven classes, and pan-drug resistance (PDR)—if antimicrobials from all but one tested classes were included in the profile (Magiorakos et al., 2012).

**Table 2:**
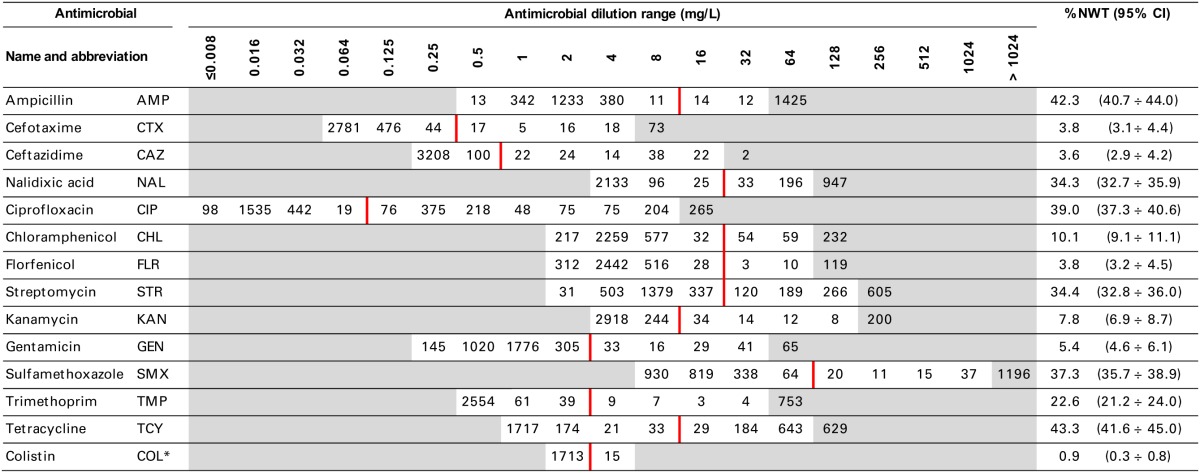
**Minimal inhibitory concentration distribution of *E. coli* isolates (*N* = 3430)**.

*White zones display the applied dilutions ranges. Vertical line indicates interpretative criteria (EUCAST epidemiological values)*.

^*^1728 isolates from 2011–12 were tested.

### Statistical analysis

**Figure 1:**
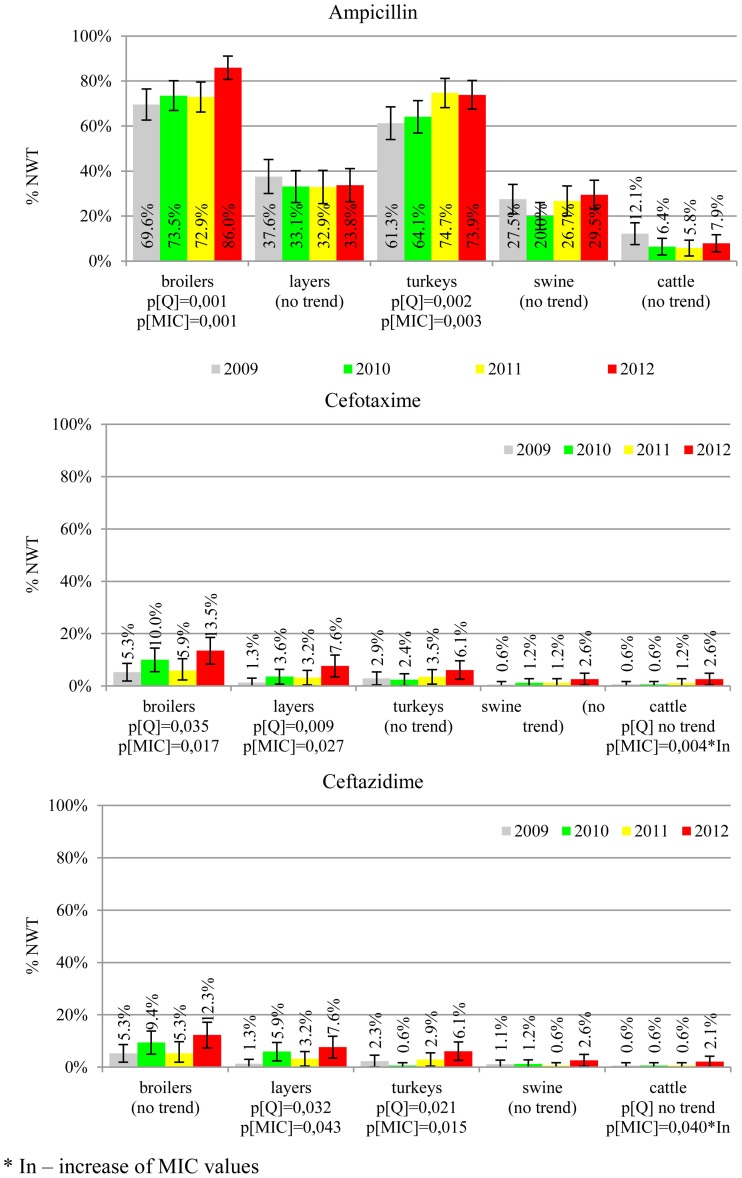
**Microbiological resistance to beta-lactams and cephalosporins**.

**Figure 2:**
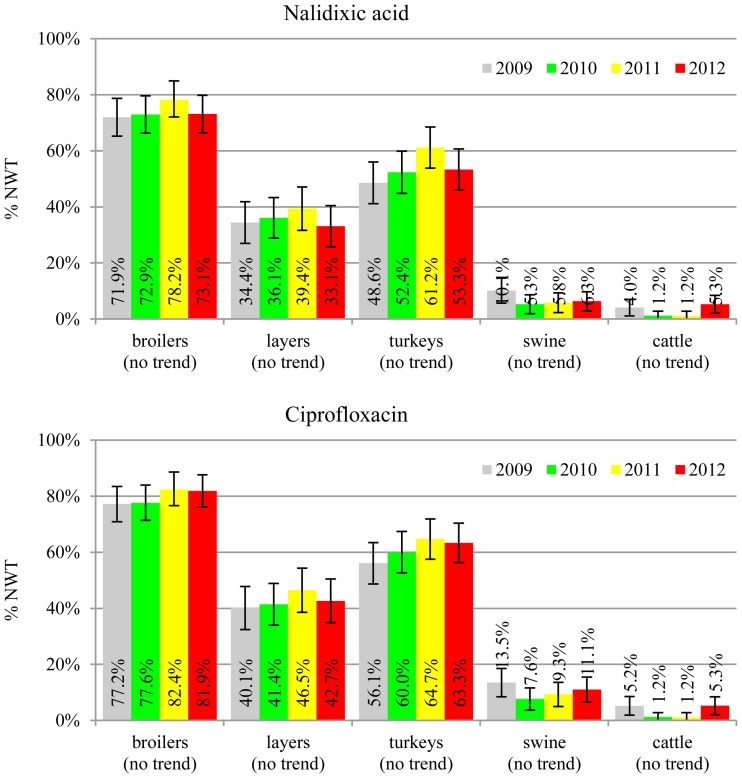
**Microbiological resistance to quinolones and fluroquinolones**.

**Figure 3:**
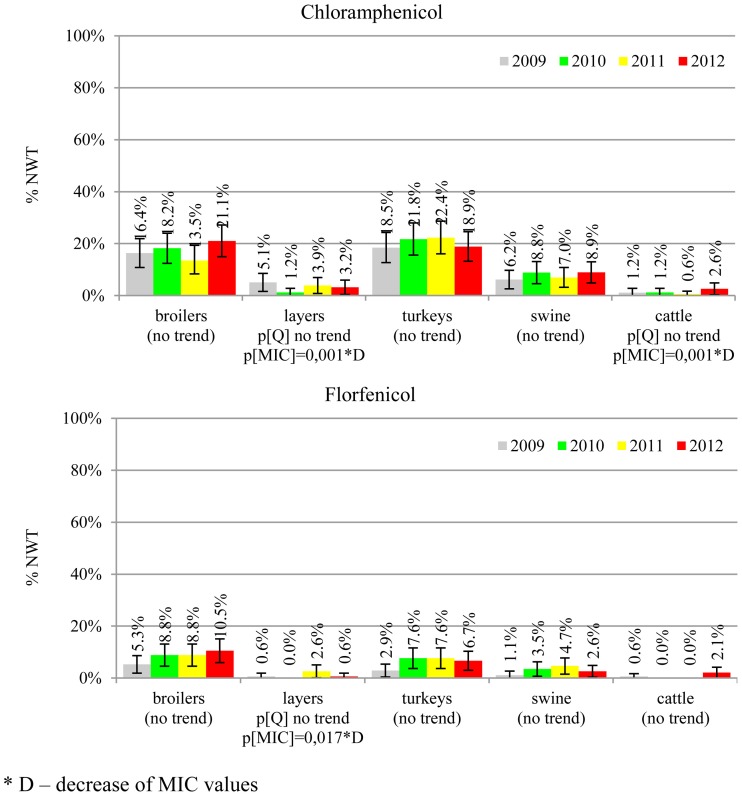
**Microbiological resistance to phenicols**.

**Figure 4:**
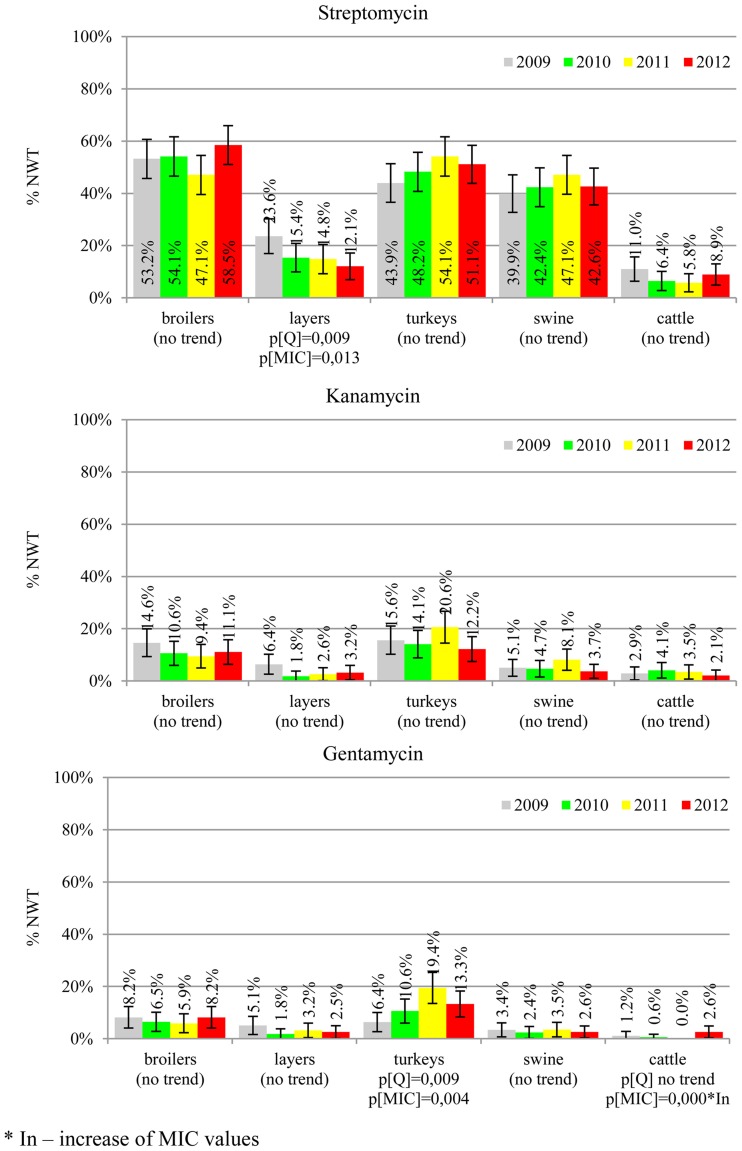
**Microbiological resistance to aminoglycosides**.

**Figure 5:**
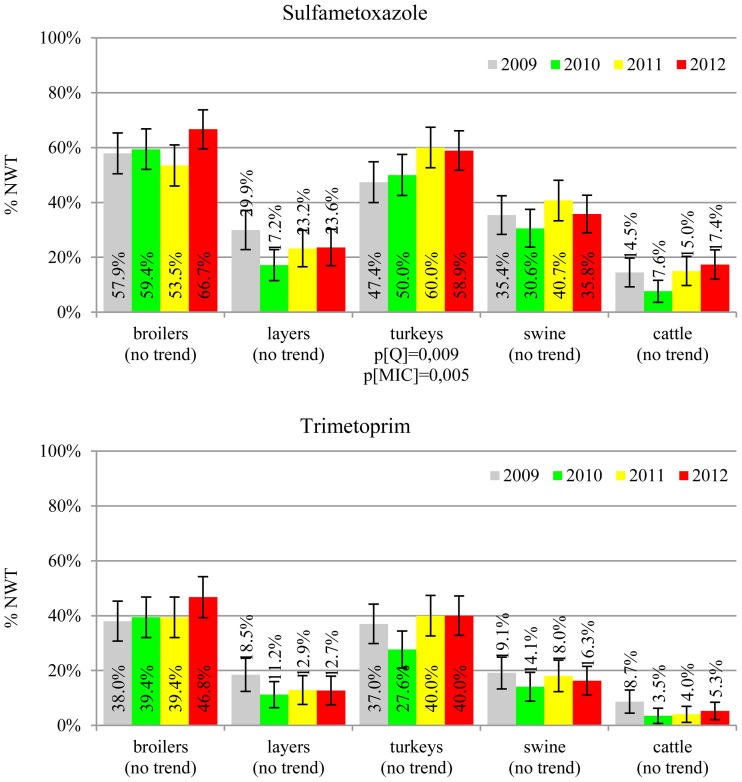
**Microbiological resistance to folate-path inhibitors**.

**Figure 6:**
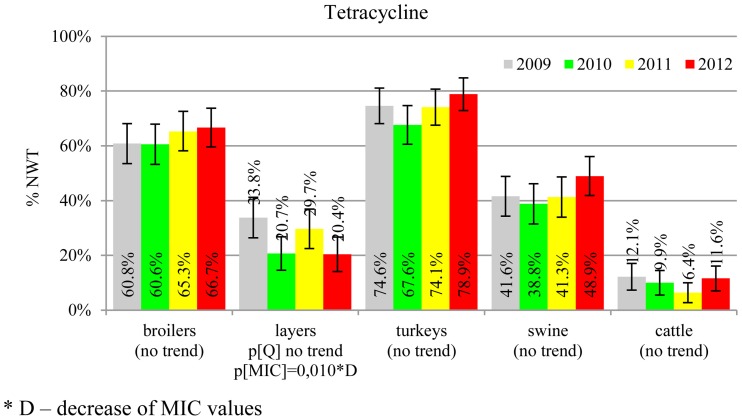
**Microbiological resistance to tetracycline**.

The prevalence of resistance was calculated as a fraction of isolates with microbiological resistance, within 95% confidence interval (95% CI). Temporal trends for each study population and all tested compounds besides colistin were assessed on qualitative and quantitative results using regression analysis (EFSA, 2012b). Qualitative results were analysed in logistic model, whereas linear regression was applied for log-2 MIC data. The *p*-values lower than 0.05 indicated significant trends graphically displayed on bar graphs (Figures 1–6).

## Results

During four-year study the number of animals slaughtered in Poland was steadily increasing although the number of slaughter plants was declining (Table 1). The adopted slaughter capacity thresholds and the numbers of selected abattoirs have been adjusted to reach finally from 70% (swine) to 99% (turkeys and laying hens) of national yearly production. The samples were collected at 439 abattoirs, on 770 (53%) days of the study period. Considering sample source (target animal), slaughterhouse ID, and sampling date, 3165 sampling events were identified and the calculated index of diversity (*D* = 0.9999) confirmed satisfactory randomization. Sampling efficacy reached 92% of 4000 planned incidents with 93.2% *E. coli* isolation rate. The target number of 170 isolates (EFSA, 2008) was achieved for all animal types but laying hens slaughtered after laying period (Table 1). As much as 70 out of 638 (11.0%) layer isolates were retrieved from animals imported from the Netherlands (*N* = 21), Germany (*N* = 17), Slovakia (*N* = 11), and Austria, Belgium, Czech Republic, Latvia and Lithuania. Imported samples were rarely (≤0.6%) recorded in other animal species but cattle.

MIC distributions of tested compounds were shown in Table 2. Microbiological resistance to all antimicrobials was identified at variable frequencies ranging from 0.9% (colistin) to 43.3% (tetracycline) but it differed considerably between antimicrobials and source of isolation (Figures 1–6). Temporal trends in prevalence of microbial resistance were found in 9 out of 65 tested antimicrobial/isolation source combinations (*p[Q]*-values shown on Figures 1–6) and in 7 further occasions MIC shifts were recorded (*p[MIC]*-values).

The average ampicillin resistance varied considerably from 75.5% in broiler isolates to 8.1% in those from cattle. Similar pattern but at several times lower frequencies was observed for cephalosporins. An increasing resistance to those antimicrobials was found in poultry isolates, but also MIC shift toward higher cephalosporin values was noted in cattle isolates (Figure 1). Nalidixic acid and ciprofloxacin resistances were observed mostly in poultry (on average at 74.0 and 79.8% in broilers, 53.8 and 61% in turkey, and 35.7 and 42.6% in layers), 6.9 and 10.4% in swine and 3.0 and 3.3% in cattle isolates. No temporal trends were found both on qualitative and quantitative results (Figure 2). The medium chloramphenicol resistance reached the highest values in turkeys and broilers (20.3 and 17.3%, respectively). Much lower levels were found in pigs (7.7%) and the lowest in laying hens (3.3%) and cattle isolates (1.4%). Florfenicol values stretched to approximately half as much in the case of chloramphenicol. Decreasing MIC values (Figure 3) were observed in layers (chloramphenicol and florfenicol) and cattle isolates (chloramphenicol). Streptomycin resistance reached the highest average values in broilers (53.2%), turkeys (49.4%), and pigs (43.0%), whereas much lower in layers (16.5%) and cattle isolates (8.1%). Kanamycin and gentamycin resistance levels were several times less frequent in comparison to streptomycin. The temporal trends analysis indicated a decrease of streptomycin resistance in laying hen isolates, an increase of gentamycin resistance in turkeys and shift in gentamycin MIC among cattle isolates (Figure 4). To exemplify the shift within wild-type population: 18% of isolates tested in 2009 showed MIC_Gen_ ≤0.25 mg/L and 39% MIC_Gen_ ≤ 1 mg/L whereas in 2012 those MIC values were noted in 2% and 47%, respectively. Sulfamethoxazole resistance was observed in up to 59.4% broiler and 54.1% turkey isolates, 35.6% in swine, 23.4% in layers and 13.7% cattle isolates. NWT trimetoprim values were generally by 10% (cattle, layers) or 20% lower than those observed for sulphonamides. The increasing trend was noted only for sulfamethoxazole in turkey isolates (Figure 5). The highest average resistance was found in turkey (73.9%) and broiler isolates (63.3%). Swine and layers isolates were resistant at 42.8% and 26.0% whereas 10.0% in cattle. Diminishing MIC values were noted in laying hens *E. coli* (Figure 6): the lowest tested concentration of 1 mg/L was found at 48% *E. coli* isolated in 2009 and 75% of the ones from the end of study period. Colistin resistance was the less frequent (Table 2), ranging from ≤0.4 to 1.6% (data not shown).

Of the 348 resistance profiles 164 were observed in the isolates originating from broilers, 111 from layers, 185 from turkeys, 120 from swine, and 56 from cattle. As much as 177 profiles were observed in single isolates, whereas 18 most frequent profiles (Table 3), each represented by at least 30 isolates, gathered 48% of *E. coli* (*N* = 1086) resistant to at least one compound. Each of them profiles tend to show up in *E. coli* originated from given animal population, i.e. Smx in cattle, Str and StrTcy in swine, NalCip in *Gallus gallus* (both broilers and layers), AmpNalCipChlStrSmxTmpTcy in slaughter poultry (broiler and turkey), or AmpNalCipStrKanSmxTmpTcy in broiler isolates. The source of *E. coli* isolation considerably influenced the complexity of resistance patterns (Table 4). MDR profiles were the least frequent in cattle (6.9 ÷ 11.2%) and approximately three times more frequent in swine and layer isolates. Up to 70.3 and 80.6% of, respectively, turkey and broiler isolates were MDR. The more complex the profiles were, the more frequently they occurred among turkey isolates: XDR and PDR profiles were at the similar frequencies found in broilers and turkeys. Two the most comprehensive profiles, each combining all tested compounds but colistin or florfenicol, were observed in *E. coli* originating from turkey (data not shown).

**Table 3:** **The 18 most common resistance patterns found in *E. coli***.

**Resistance pattern**	No [%^*^] of isolates					
	**Broilers**	**Layers**	**Turkeys**	**Swine**	**Cattle**	**Total**
AmpNalCipStrSmxTmpTcy	64 [47%]	14	51 [38%]	4	3	136
NalCip	40 [31%]	65 [50%]	22	2	2	131
Tcy	8	12	29	48 [44%]	13	110
AmpNalCipTcy	31 [37%]	17	32 [39%]	2	1	83
Smx	4	15	11	14	33 [43%]	77
Amp	11	22 [37%]	2	17	7	59
StrTcy		4	4	42 [75%]	6	56
AmpTcy	11	12	24 [44%]	6	1	54
AmpNalCip	21 [43%]	16 [33%]	11	1		49
AmpStrSmxTmpTcy	14 [32%]	3	7	17 [39%]	3	44
StrSmxTcy	1		1	34 [79%]	7	43
AmpNalCipChlStrSmxTmpTcy	16 [39%]	4	21 [51%]			41
AmpNalCipStrTcy	16 [42%]	7	12 [32%]	3		38
Str	2		1	30 [88%]	1	34
AmpNalCipStrSmxTcy	17 [50%]	5	10	2		34
AmpNalCipSmxTmpTcy	25 [74%]	3	6			34
AmpStrSmxTcy	9		7	14 [42%]	3	33
AmpNalCipStrKanSmxTmpTcy	17 [57%]	1	9	2	1	30

* *Percentage of the isolates representing a profile is shown if ≥ 30%*.

**Table 4:** **Complexity of resistance patterns by source of *E. coli* isolation**.

**Resistance patterns**		**Source of *E. coli* isolation (No of tested isolates)**					
		**Broilers (682)**	**Layers (638)**	**Turkeys (693)**	**Swine (710)**	**Cattle (707)**	**Total (3430)**
Wild-type (no resistance)	%	5.1%	39.3%	11.3%	35.9%	79.9%	34.5%
	95% CI	3.5 ÷ 6.8%	35.6 ÷ 43.1%	8.9 ÷ 13.6%	32.4 ÷ 39.4%	77.0 ÷ 82.9%	32.9 ÷ 36.1%
Multi-drug resistance (≥3 antimicrobial classes)	%	80.6%	31.0%	70.3%	33.7%	9.1%	44.8%
	95% CI	77.7 ÷ 83.6%	27.4 ÷ 34.6%	66.9 ÷ 73.7%	30.2 ÷ 37.1%	6.9 ÷ 11.2%	43.2 ÷ 46.5%
Extensive-drug resistance (7 ÷ 9 antimicrobial classes)	%	11.7%	3.3%	13.3%	0.8%	0.3%	5.9%
	95% CI	9.3 ÷ 14.1%	1.9 ÷ 4.7%	10.7 ÷ 15.8%	0.2 ÷ 1.5%	0.0 ÷ 0.7%	5.1 ÷ 6.6%
Pan-drug resistance (8 ÷ 9 antimicrobial classes)	%	1.6%	0.2%	1.9%	0.1%	0.0%	0.8%
	95% CI	0.7 ÷ 2.6%	0.0 ÷ 0.5%	0.9 ÷ 2.9%	0.0 ÷ 0.4%	0.0 ÷ 0.0%	0.5 ÷ 1.0%

## Discussion

Multiple resistance monitoring systems have been introduced world-wide (Bronzwaer et al., 2008; Deckert et al., 2010; Ozaki et al., 2011; Morfin-Otero et al., 2012; Tadesse et al., 2012). Current study, although sampling involved only a sparse fraction of animals slaughtered in the country, due to harmonized methodology and comparable numbers of random isolates collected regularly over the study period (SVARM, 2012; Tadesse et al., 2012) offers direct comparison between isolates from different sources, geographical locations and time frames (MARAN, 2012; Schroeter et al., 2013). The use of commensal intestinal *E. coli* as indicator for the presence of resistance determinants in bacterial flora is considered a key component of surveillance programs both in food-producing animals and wildlife (De Jong et al., 2012; EFSA and ECDC, 2012; SVARM, 2012; Allen et al., 2013). The frequency of resistance is considered a marker of selection pressure exerted by antimicrobial use in the host animal population (MARAN, 2012; SVARM, 2012; Allen et al., 2013). Resistant isolates found in current study in samples collected from imported animals (data not shown) indicate animal trade as a vector of resistance dissemination (Wasyl et al., 2012). The reflection of antimicrobial usage policies in different animal husbandry has been noted in current results. Most (79.9%) of cattle *E. coli* showed no resistance (Table 4). Since those isolates originated mostly from adult cattle (medium age at slaughter: 46 months, max. 214 months; data not shown) it might be presumed that some animals were slaughtered due to insufficient milk productivity and had not been treated with antimicrobials due to restriction on milk during withdrawal period. On the opposite, poultry might be treated until few days before slaughter and thus high resistance levels were found: only 5.1% of broiler and 11.3% of turkey isolates showed no resistance (Table 4). Some correlation between source of isolation and resistance profile complexity was noted. The most relevant one was increasing number of extensively and pan-drug resistances in turkey isolates compared to broilers. Those public health relevant resistances (Magiorakos et al., 2012) were rarely detected in isolates from layers, pigs and cattle. The antimicrobial consumption data support that observation as well as some resistance pattern overlap with preferences of antimicrobial usage in different animal species (EMA, 2012; Krasucka et al., 2012). Low prevalence of resistant *E. coli* in adult cattle or frequent tetracycline, penicillins, sulphonamides, and trimetoprim resistances in pig isolates were also reported from other countries (MARAN, 2012; SVARM, 2012). Finding of specific resistance profiles in isolates originating from defined animal population (Table 3) might further support the selective effect of antimicrobial usage. Based on broad time and geographical sampling frame as well as PFGE typing of a selection the isolates (data not shown), the clonal spread of certain resistance phenotypes might be neglected.

Interpretation criteria as an essential part of resistance monitoring should also be addressed. Epidemiological cut-off values are often criticized by clinicians (De Jong et al., 2012). Herewith it has been proved, that besides being appropriate for the isolates originating from non-diseased animals, current approach gives a great opportunity for temporal trend analysis. Resistance trend analysis is often made on interpreted data (Kronvall, 2010; Kaesbohrer et al., 2012; Morfin-Otero et al., 2012; SVARM, 2012; Tadesse et al., 2012; Van Der Bij et al., 2012). The advantages of quantitative analysis of MIC values have been recently raised (EFSA, 2012a). To our knowledge, present study is one of the few full-scale MIC distributions explored with trend analysis (Lundin et al., 2008). Increasing trends observed both on qualitative and quantitative level were detected in poultry isolates being resistant to beta-lactams, cephalosporins, aminoglycosides, and sulphametoxazole. Analysis on MIC shifts gave possibility for early detection of resistance trends (Lundin et al., 2008). Surprisingly, decreasing MIC values were found in layers isolates for chloramphenicol, florfenicol, and tetracycline, as well as chloramphenicol in *E. coli* from cattle. That finding is in disagreement to the general perception of antimicrobial resistance as emerging problem (Collignon et al., 2009) and should be of relevance for clinical surveys (De Jong et al., 2012).

Even though no temporal trends were noted in quinolone and fluoroquinolone resistance, its prevalence in poultry isolates is worrisome (Figure 2). The MIC values higher than clinical breakpoint (≥0.5 mg/L) found in 19.4% of non-pathogenic isolates (Table 2) indicate potential clinical consequences. The compounds are considered critically important for human medicine (Collignon et al., 2009), but the mechanisms behind, namely step-wise chromosomal resistance due to gyrase and topoisomerase IV genes mutations, may not be shared between bacteria (Hordijk et al., 2012). Plasmid mediated resistance was less common, but of possible importance (EFSA, 2008; Veldman et al., 2011). The 4.7% difference between microbiological resistance to ciprofloxacin and nalidixic acid might indicate the scale of the problem that peaked up to 8.8, 9.6 and 10.0% in, respectively, broiler, layer and turkey *E. coli* isolated in 2012. Such high occurrence of plasmid mediated determinants in Poland has already been reported (Veldman et al., 2011).

*E. coli* resistance to cephalosporins results from various transmissible and chromosomally encoded mechanisms (Dahmen et al., 2012; Wasyl et al., 2012). The observed levels of cefotaxime (3.8%) and ceftazidime (3.6%) NWT MICs (Table 2) indicate on both animal reservoirs and imperfection of cephalosporin resistance screening in random commensal *E. coli*. The previous studies showed that selective screening might result in 10-fold increase of isolates conferring ESBL or ampC-type resistance (Wasyl et al., 2010). Interestingly, the resistance determinants differed from the ones found in humans (Empel et al., 2008; Dahmen et al., 2012; Wasyl et al., 2012). Resistance to ampicillin, ranked the second highest (43.3%) and reaching 86.0% of broiler isolates in 2012, may also compromise critical importance in human and animal use. Such high prevalence might result from both long period of application of the old antimicrobial class, and co-selection of resistance mechanisms by other compounds. Remarkably, the resistances are very dynamic since 8 out of 12 increasing trends were noted in beta-lactams and cephalosporins (Figure 1). Some dynamics were observed also in tetracyclines (decreasing MIC values in layer isolates) and sulfametoxazole (increasing resistance in turkeys isolates). Those compounds, including sulphonamides combinations with trimethoprim, have been used for decades and are still of clinical importance (EFSA, 2008; Pedersen et al., 2009; Van Der Bij et al., 2012) although resistance has usually ranked amongst the highest both in human and animal *E. coli* (Deckert et al., 2010; De Jong et al., 2012; Kaesbohrer et al., 2012; SVARM, 2012; Tadesse et al., 2012). Therapeutic applications of colistin, another decades-old antimicrobial, was limited due to high toxicity. Currently it has been re-discovered as a last resort drug for control of multidrug resistant gram-negative bacteria (Collignon et al., 2009; Pogue et al., 2011). The observed resistance (0.9%) was found either alone or as MDR component (data not shown) and should be considered highly important (Collignon et al., 2009). The aforementioned levels and trends of resistance might suggest, that the concept of higher resistance against older antimicrobials compared to newly introduced compounds (Tadesse et al., 2012) is not entirely justified.

Streptomycin has been used extensively in animals and resistance is common in commensal and pathogenic *E. coli* (Hendriksen et al., 2008; Pedersen et al., 2009; Stannarius et al., 2009; SVARM, 2012; Tadesse et al., 2012). Though the resistance testing is tricky and multiple genes are involved, the compound is useful epidemiological marker for various resistance phenotypes, and it should be considered important besides minor clinical importance of streptomycin in humans (EFSA, 2008; Collignon et al., 2009). Noteworthy, layer isolates revealed the only decreasing trend on interpreted data found in current study (Figure 4). The remaining aminoglycosides showed much less resistance, with prevalence comparable to other countries and the highest values observed in turkey isolates (Kaesbohrer et al., 2012; MARAN, 2012; SVARM, 2012; Tadesse et al., 2012).

Different levels of chloramphenicol (10.1%) and florfenicol (3.8%) resistance allowed at rough differentiation of background mechanisms (Bronzwaer et al., 2008; EFSA, 2008) commonly associated with mobile genetic elements playing major role in dissemination of multiple antimicrobial drug resistance (Tadesse et al., 2012). Chloramphenicol resistance can smoothly evolve under selective pressure (Toprak et al., 2011), but presumably due to its ban for animal use, a diminishing MIC values were observed in layer and cattle isolates (Figure 3). Resistance to florfenicol, a veterinary used derivative remains low and might even decrease.

Our study has shown that antimicrobial resistance is an ever evolving issue driven by antimicrobial usage pressure. It was proved by variable resistances observed in commensal *E. coli* from different slaughter animals and increasing resistance trends observed in several occasions. However, decrease in proportion of microbiologically resistant isolates, as well as diminishing MIC values indicate on complex and multivariable aspects of resistance. Definitely *E. coli* may act as reservoir of resistance genes for other bacteria, including pathogenic bacteria (Veldman et al., 2011; Dahmen et al., 2012; Wasyl et al., 2012). Current results might be considered reliable background for assessment of effects of antimicrobial usage in animal and indicate the needs and areas for in-depth research on resistance mechanisms, their development and spread

### Conflict of interest statement

The authors declare that the research was conducted in the absence of any commercial or financial relationships that could be construed as a potential conflict of interest.

## References

[B1] Allen S. E. Janecko N. Pearl D. L. Boerlin P. Reid-Smith R. J. Jardine C. M. (2013). Comparison of *Escherichia coli* recovery and antimicrobial resistance in cecal, colon, and fecal samples collected from wild house mice (*Mus musculus*). J. Wildl. Dis. 49, 432–436 10.7589/2012-05-142 23568923

[B2] Bronzwaer S. Aarestrup F. Battisti A. Bengtsson B. Piriz Duran S. Emborg H.-D. (2008). Harmonised monitoring of antimicrobial resistance in *Salmonella* and *Campylobacter* isolates from food animals in the European Union. Clin. Microbiol. Infec. 14, 522–533 10.1111/j.1469-0691.2008.02000.x 18397331

[B3] Collignon P. Powers J. H. Chiller T. M. Aidara-Kane A. Aarestrup F. M. (2009). World Health Organization ranking of antimicrobials according to their importance in human medicine: a critical step for developing risk management strategies for the use of antimicrobials in food production animals. Clin. Infect. Dis. 49, 132–141 10.1086/599374 19489713

[B4] Dahmen S. Haenni M. Madec J. Y. (2012). IncI1/ST3 plasmids contribute to the dissemination of the blaCTX-M-1 gene in *Escherichia coli* from several animal species in France. J. Antimicrob. Chemother. 67, 3011–3012 10.1093/jac/dks308 22872449

[B5] Deckert A. Gow S. Rosengren L. Leger D. Avery B. Daignault D. (2010). Canadian integrated program for antimicrobial resistance surveillance (CIPARS) farm program: results from finisher pig surveillance. Zoonoses Public Health 57, 71–84 10.1111/j.1863-2378.2010.01356.x 21083820

[B6] De Jong A. Thomas V. Simjee S. Godinho K. Schiessl B. Klein U. (2012). Pan-European monitoring of susceptibility to human-use antimicrobial agents in enteric bacteria isolated from healthy food-producing animals. J. Antimicrob. Chemother. 67, 638–651 10.1093/jac/dkr539 22210758

[B7] EFSA. (2008). Report from the task force on zoonoses data collection including guidance for harmonized monitoring and reporting of antimicrobial resistance in commensal *Escherichia coli* and *Enterococcus* spp. from food animals. EFSA J. 141, 1–44

[B8] EFSA. (2009). Joint opinion on antimicrobial resistance (AMR) focused on zoonotic infections. Scientific opinion of the European Centre for Disease Prevention and Control; Scientific Opinion of the Panel on Biological Hazards; Opinion of the Committee for Medicinal Products for Veterinary Use; Scientific Opinion of the Scientific Committee on Emerging and Newly Identified Health Risks. EFSA J. 7, 1–78

[B9] EFSA. (2012a). Technical specifications for the analysis and reporting of data on antimicrobial resistance in the European Union summary report. EFSA J. 10, 1–53

[B10] EFSA. (2012b). Technical specifications on the harmonised monitoring and reporting of antimicrobial resistance in *Salmonella*, *Campylobacter* and indicator *Escherichia coli* and *Enterococcus* spp. bacteria transmitted through food. EFSA J. 10, 1–64

[B11] EFSA ECDC. (2012). The European Union Summary report on antimicrobial resistance in zoonotic and indicator bacteria from humans, animals and food in 2010. EFSA J. 10, 1–233

[B12] EMA. (2012). Sales of Veterinary Antimicrobial Agents in 19 EU/EEA Countries in 2010. ESVAC report EMA/88728/2012, London.

[B13] Empel J. Baraniak A. Literacka E. Mrowka A. Fiett J. Sadowy E. (2008). Molecular survey of beta-lactamases conferring resistance to newer beta-lactams in enterobacteriaceae isolates from Polish hospitals. Antimicrob. Agents Chemother. 52, 2449–2454 10.1128/AAC.00043-08 18458126 PMC2443902

[B14] Hendriksen R. S. Mevius D. J. Schroeter A. Teale C. Meunier D. Butaye P. (2008). Prevalence of antimicrobial resistance among bacterial pathogens isolated from cattle in different European countries: 2002-2004. Acta Vet. Scand. 50, 28 18611246 10.1186/1751-0147-50-28 PMC2486267

[B15] Hordijk J. Veldman K. Dierikx C. Van Essen-Zandbergen A. Wagenaar J. A. Mevius D. (2012). Prevalence and characteristics of quinolone resistance in *Escherichia coli* in veal calves. Vet. Microbiol. 156, 136–142 10.1016/j.vetmic.2011.10.006 22041448

[B16] Kaesbohrer A. Schroeter A. Tenhagen B. A. Alt K. Guerra B. Appel B. (2012). Emerging antimicrobial resistance in commensal *Escherichia coli* with public health relevance. Zoonoses Public Health 59, 158–165 10.1111/j.1863-2378.2011.01451.x 22958260

[B17] Krasucka D. Cybulski W. Klimowicz A. (2012). Ocena stosowania substancji przeciwdrobnoustrojowych u świń i bydła w Polsce na podstawie badań sondażowych w 2010 roku. Medycyna Weterynaryjna 68, 106–109

[B18] Kronvall G. (2010). Antimicrobial resistance 1979-2009 at Karolinska hospital, Sweden: normalized resistance interpretation during a 30-year follow-up on *Staphylococcus aureus* and *Escherichia coli* resistance development. APMIS 118, 621–639 10.1111/j.1600-0463.2010.02660.x 20718714

[B19] Lundin J. I. Dargatz D. A. Wagner B. A. Lombard J. E. Hill A. E. Ladely S. R. (2008). Antimicrobial drug resistance of fecal *Escherichia coli* and *Salmonella* spp. isolates from United States dairy cows. Foodborne Pathog. Dis. 5, 7–19 10.1089/fpd.2007.0018 18260811

[B20] Magiorakos A. P. Srinivasan A. Carey R. B. Carmeli Y. Falagas M. E. Giske C. G. (2012). Multidrug-resistant, extensively drug-resistant and pandrug-resistant bacteria: an international expert proposal for interim standard definitions for acquired resistance. Clin. Microbiol. Infect. 18, 268–281 10.1111/j.1469-0691.2011.03570.x 21793988

[B21] MARAN. (2012). Monitoring of Antimicrobial Resistance and Antibiotic Usage in Animals in the Netherlands. Wageningen, Central Veterinary Institute, Agricultural Economics Research Institute Available online at: www.maran.wur.nl

[B22] Morfin-Otero R. Tinoco-Favila J. C. Sader H. S. Salcido-Gutierrez L. Perez-Gomez H. R. Gonzalez-Diaz E. (2012). Resistance trends in gram-negative bacteria: surveillance results from two Mexican hospitals, 2005-2010. BMC Res. Notes 5:277 10.1186/1756-0500-5-277 22676813 PMC3407022

[B23] Ozaki H. Esaki H. Takemoto K. Ikeda A. Nakatani Y. Someya A. (2011). Antimicrobial resistance in fecal *Escherichia coli* isolated from growing chickens on commercial broiler farms. Vet. Microbiol. 150, 132–139 10.1016/j.vetmic.2010.12.020 21232883

[B24] Pedersen K. Hammer A. S. Sorensen C. M. Heuer O. E. (2009). Usage of antimicrobials and occurrence of antimicrobial resistance among bacteria from mink. Vet. Microbiol. 133, 115–122 10.1016/j.vetmic.2008.06.005 18620819

[B25] Perchec-Merien A. M. Lewis G. D. (2013). Naturalized *Escherichia coli* from New Zealand wetland and stream environments. FEMS Microbiol. Ecol. 83, 494–503 10.1111/1574-6941.12010 22974403

[B26] Pitout J. D. (2012). Extraintestinal pathogenic *Escherichia coli*: a combination of virulence with antibiotic resistance. Front. Microbiol. 3:9 10.3389/fmicb.2012.00009 22294983 PMC3261549

[B27] Pogue J. M. Marchaim D. Kaye D. Kaye K. S. (2011). Revisiting “older” antimicrobials in the era of multidrug resistance. Pharmacotherapy 31, 912–921 10.1592/phco.31.9.912 21923592

[B28] Schroeter A. Tenhagen B.-A. Alt K. Fetsch A. Stingl K. Guerra B. (2013). German Antimicrobial Resistance Situation in the Food Chain – Darlink 2009. Berlin: BfR Wissenschaft

[B29] Stannarius C. Burgi E. Regula G. Zychowska M. A. Zweifel C. Stephan R. (2009). Antimicrobial resistance in *Escherichia coli* strains isolated from Swiss weaned pigs and sows. Schweizer Archiv für Tierheilkunde 151, 119–125 10.1024/0036-7281.151.3.119 19263381

[B30] SVARM. (2012). Swedish Veterinary Antimicrobial Resistance Monitoring. Uppsala, Sweden: The National Veterinary Institute (SVA)

[B31] Tadesse D. A. Zhao S. Tong E. Ayers S. Singh A. Bartholomew M. J. (2012). Antimicrobial drug resistance in *Escherichia coli* from humans and food animals, United States, 1950-2002. Emerg. Infect. Dis. 18, 741–749 10.3201/eid1805.111153 22515968 PMC3358085

[B32] Toprak E. Veres A. Michel J. B. Chait R. Hartl D. L. Kishony R. (2011). Evolutionary paths to antibiotic resistance under dynamically sustained drug selection. Nat. Genet. 44, 101–105 10.1038/ng.1034 22179135 PMC3534735

[B33] Van Der Bij A. K. Van Dijk K. Muilwijk J. Thijsen S. F. Notermans D. W. De Greeff S. (2012). Clinical breakpoint changes and their impact on surveillance of antimicrobial resistance in *Escherichia coli* causing bacteraemia. Clin. Microbiol. Infect. 18, E466–E472 22925456 10.1111/j.1469-0691.2012.03996.x

[B34] Veldman K. Cavaco L. M. Mevius D. Battisti A. Franco A. Botteldoorn N. (2011). International collaborative study on the occurrence of plasmid-mediated quinolone resistance in *Salmonella enterica* and *Escherichia coli* isolated from animals, humans, food and the environment in 13 European countries. J. Antimicrob. Chemother. 66, 1278–1286 10.1093/jac/dkr084 21393198

[B35] Wasyl D. Hasman H. Cavaco L. M. Aarestrup F. M. (2012). Prevalence and characterization of cephalosporin resistance in nonpathogenic *Escherichia coli* from food-producing animals slaughtered in Poland. Microb. Drug Resist. 18, 79–82 10.1089/mdr.2011.0033 21721933

[B36] Wasyl D. Hoszowski A. Zając M. Skarżyñska M. (2010). Simple and efficient screening method for detection of cephalosporin resistant *Escherichia coli*. Bull. Vet. Inst. Pulawy 54, 147–151

